# A Terrain Elevation Map Generation Method Based on Self-Attention Mechanism and Multifeature Sketch

**DOI:** 10.1155/2022/9481445

**Published:** 2022-03-29

**Authors:** Xingquan Cai, Mengyao Xi, Nu Yu, Zhe Yang, Haiyan Sun

**Affiliations:** School of Information Science and Technology, North China University of Technology, Beijing 100144, China

## Abstract

To address the issues of low efficiency in manual terrain feature map annotating and poor realism in terrain elevation map generation, this paper proposes a terrain elevation map generation method based on self-attention mechanism and multifeature sketch. Firstly, the proposed method extracts features from a terrain elevation map using an adaptive feature enhancement method. Afterwards, our method adds a self-attention mechanism to the generator and discriminator of conditional generative adversarial network to capture the global spatial features and generates a realistic terrain elevation map. Finally, a level of detail method is used to visualize the three-dimensional terrain, and an interactive terrain editing tool for roaming interaction is implemented. Experimental data show that the proposed method performs well in subjective visual performance and objective criteria and has obvious advantages over other current typical methods.

## 1. Introduction

Terrain is one of the most common elements in nature. Realistic 3D terrains have been widely used in 3D video games, military simulations, film industry, and other fields. For instance, developers in 3D video games can create a game world that brings immersive user experience to players; realistic battlefields can be simulated for virtual military training in military simulations; visual effect artists can create scenes with realistic terrains that bring amazing visual experience to the audience in the film industry. Hence, digital terrains with realistic features can play an important role in enhancing the realism of virtual scenes and improving user immersion.

Interactive terrain editing tools aim at generating realistic terrains efficiently based on the sketch input from users. These editing tools should provide rich functionalities that can support users with a little technical background. In terms of realism, generated terrains should have natural features of the terrain in the real world, where no traces of manual editing can be found. Since interactive terrain editing tools can be widely used in various applications, it is important to investigate how to efficiently generate terrain elevation maps with realistic natural features.

In recent years, many researchers have investigated terrain editing. Existing terrain editing methods can be classified into three categories: terrain editing based on fractal noises, terrain editing based on physical erosion simulations, and terrain editing based on real samples. Specifically, for terrain editing methods based on fractal noises, a terrain is simulated by generating fractal noises based on user input parameters. The process is hardly controllable, which is difficult for users to get results that meet their demands. As for the methods based on physical erosion simulations, users need to edit a terrain with the knowledge of physical erosion theory, which is difficult for nonprofessional users. Finally, for the methods based on real samples, despite the fact that generated terrains can retain some natural features of real terrain, there is still much room for optimization in terms of realism and interaction experience. In order to enable users to perform interactive editing flexibly and generate realistic terrain, this paper draws on the methods based on real samples, which not only ensures that the generated results have the features of real samples but also enhances the realism of the generated results further. At the same time, the proposed method can improve computing efficiency, meet real-time interaction requirements, and enhance the user's interactive experience by visualizing the three-dimensional terrain. Therefore, it is necessary to investigate interactive terrain editing techniques based on multifeature sketches.

The contributions are summarized as follows:

1. We propose a terrain feature extraction method based on adaptive feature enhancement to solve the problem of low efficiency of manual annotation.
2. We propose a terrain elevation map generation method based on self-attention mechanism. The conditional generative adversarial network is used to generate a realistic terrain elevation map.
3. We propose a three-dimensional terrain visualization method based on Levels of Detail, which improves the fluency of user interaction.
4. We realize an interactive terrain editing tool based on multifeature sketches. The tool runs stably and has a good user interaction experience.

## 2. Related Work

Interactive terrain editing tools can generate terrain elevation maps based on various terrain features from user input. Relevant scholars proposed a method for interactive generation and display of 3D terrain [1]. The method can automatically generate terrain that meets the user's needs, but the feedback of the interactive operation is yet to be studied. The researchers gradually generated the terrain by expanding the details of the user sketch [2]. The algorithm is computationally efficient and highly interactive. Related scholars used terrain elevation entropy, extracted features from Digital Elevation Model (DEM) data to generate terrain frames, and accelerated mapping using GPU to improve the efficiency of terrain generation during the editing of terrain [3]. Given the high efficiency of the GPU, a terrain editing algorithm based on CUDA architecture was proposed [4], which is applicable to large-scale terrain scene creation. With the development of deep learning, the researchers applied this to the terrain editing problem and proposed a real-time interactive terrain editing method [5]. The results obtained by this method conform to the feature distribution of real terrain samples with high fidelity. Related scholars implemented interactive terrain editing by providing the user with functions related to plate tectonics as well as erosion simulation [6]. The results of terrain editing obtained by this method are in accordance with the theories related to geosciences and are visually closer to the real terrain. Researchers have proved that the approach with machine learning could enhance the realism of procedurally generated terrain [7]. The method used Deep Convolutional Generative Adversarial Network (DCGAN) to generate height maps, and the generated results outperformed the noise-generated elevation map results, achieving a more realistic terrain elevation map editing. The authors proposed a generation method based on implicit feature representation of terrain [8]. This method can create large-scale terrain with less memory consumption and can implement special terrain. To solve the problem of less efficient manual editing, a semiautomatic procedural terrain generation method for terrain editing tasks has been proven to work [9].

Specifically, terrain feature extraction method, terrain elevation map generation method, and terrain visualization method will be introduced as follows.

For terrain feature extractions, the primary goal is to extract ridge lines and valley lines efficiently and accurately. A Profile recognition and Polygon breaking Algorithm (PPA) was firstly proposed in [10]. This method can efficiently extract terrain ridge lines and valley lines that were consistent with the actual observation. The researchers proposed the necessity of effectively selecting terrain elevation profiles based on DEM data, which can improve the accuracy of traditional elevation profile extraction methods [11]. Based on the research [10], relevant scholars proved that morphological correlation algorithm could be used to complete the region refinement after polygon construction [12]. Related researchers proposed a terrain feature extraction algorithm to control feature significance, which can obtain extraction results of terrain feature lines that were in line with human eye observations [13]. Inspired by PPA, we optimize the process of removing redundant branches and leaves in a feature-connected graph when extracting ridge lines and valley lines. We propose a terrain feature extraction method based on adaptive feature enhancement, which can extract ridge lines, valley lines, and peak points in large-scale data set production tasks.

For terrain elevation map generations, deep learning algorithms become mainstream in the field of image generation. Generative Adversarial Network was proposed [14], which can create a model for generating high-definition images. Subsequently, the researchers proposed Conditional Generative Adversarial Network that added constraints to the Generative Adversarial Network, which can use category labels as a condition to get generation results [15]. The scholars proposed Deep Convolutional Generative Adversarial Network (DCGAN) [16], which introduced convolutional operations into the structure of Generative Adversarial Network for the first time. This method can capture the features of input data well and realize supervised learning. Based on the research in [15], related scholars proposed a method where an input image can be mapped to an output image using a Conditional Generative Adversarial Network, which can effectively convert picture labels to pictures [17]. The researchers added a self-attention mechanism to the network's construction, which can capture structural global features more easily [18]. Inspired by the work [17, 18], we apply a Conditional Generative Adversarial Network in terrain elevation map generation tasks. In addition, a self-attention mechanism is used to capture the global structure of terrain feature map, which can improve the performance of generator and discriminator to generate realistic terrains.

For terrain visualizations, terrain mesh generation and texture mapping are two key issues. For terrain mesh generation, it is mainly based on Levels of Detail (LOD) techniques. Related scholars proposed the concept of LOD for the first time and proposed a method for reducing the complexity of model mesh in a recursive way [19]. The researchers introduced elevation variation coefficient to describe a terrain, which can improve the rendering efficiency of LOD algorithm when constructing a terrain mesh [20]. Relevant researchers proposed the layer-based Discrete Cosine Transform (DCT) method to simplify the terrain data in the terrain grid construction process, improving the efficiency of terrain grid construction [21]. For texture mapping, the researchers proposed a texture mapping method with multifeature control, which considered both terrain elevation value and terrain slope [22]. Researchers proposed a multiresolution texture seamless mapping method based on error control, which can realize seamless mapping by reducing projection errors based on the distance between texture block and viewpoint and the current LOD level [23]. Related scholars proved that a beach scene simulation method based on Poisson fusion, which can not only ensure realism but also effectively improve the rendering efficiency [24]. In order to build terrain mesh efficiently and improve the efficiency of terrain rendering, this paper adopts the terrain visualization method based on Levels of Detail.

## 3. Terrain Elevation Map Generation Based on Self-Attention Mechanism and Multifeature Sketches

To generate terrain elevation maps with rich natural features that can improve user immersion, our method first extracts terrain features based on adaptive feature enhancement to obtain terrain feature maps, then generates realistic terrain elevation maps based on self-attention mechanism, and finally realizes 3D terrain visualization based on Levels of Detail.

### 3.1. Terrain Feature Extraction Based on Adaptive Feature Enhancement

When generating terrain elevation maps, a large number of terrain samples and feature maps are needed as data sets. However, methods based on manual marking terrain feature maps are inefficient and cannot meet the practical application requirements. To extract terrain features efficiently and generate terrain feature maps automatically, it is necessary to study terrain feature extraction algorithms based on adaptive feature enhancement. In our method, an adaptive feature enhancement algorithm is used to preprocess the input data first. Afterwards, ridge lines, valley lines, and peak points are extracted by a profile recognition method. Finally, these terrain features can be extracted quickly and accurately as a terrain feature map.

#### 3.1.1. Self-Adaptive Feature Enhancement

When performing adaptive feature enhancement, data need to be preprocessed first, followed by grayscale expansion. In the data preprocessing stage, when a terrain elevation map is input, the gray histogram is constructed.

As the larger the range of image grayscale, the difference between images will be more obvious, and more features can be distinguished. Hence, it is necessary to expand the grayscale. The detailed steps of image grayscale expansion are as follows:

*Step 1*. Pixel number calculation: our method traverses the horizontal axis of a gray histogram in sequential order and then in reverse order to accumulate the number of pixels.
*Step 2*. Grayscale expansion: our method calculates the percentage of accumulated pixels to total pixels and sets a threshold *K*_gray_. When this percentage reaches *K*_gray_, the method records the minimum and maximum grayscale values as *I*_min_ and *I*_max_, respectively. Pixel values that are less than *I*_min_ are mapped to 0 and pixel values that are greater than *I*_max_ are mapped to 255, as shown in (1):
  (1) $$\begin{array}{c} O\left(i,j\right)=\left\{\begin{array}{cc} 0, & I\left(i,j\right)<I_{\min}\\ 255, & I\left(i,j\right)>I_{\max} \end{array}\right., \end{array}$$
where *I*(*i*, *j*) is the pixel value for row *i*, column *j* of the input image; *O*(*i*, *j*) is the pixel value for row *i*, column *j* of the output image.
*Step 3*. Histogram equalization: the method performs histogram equalization for those pixels that have a grayscale value between *I*_min_ and *I*_max_ using (2), so that the pixels of each grayscale can be evenly distributed.

(2) $$\begin{array}{c} O\left(i,j\right)=255*\frac{I\left(i,j\right)-I_{\min}}{I_{\max}-I_{\min}}. \end{array}$$

**Figure 1:**
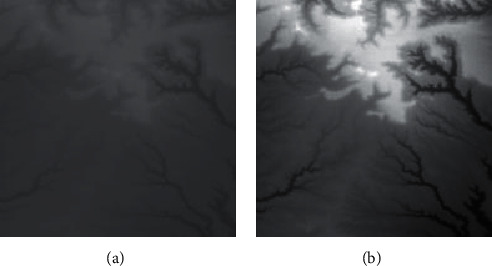
Comparisons of the effects before and after adaptive feature enhancement calculation. (a) Input image. (b) Output image.

The effect of grayscale expansion is shown in Figure 1, where the input image is shown in Figure 1(a), and the output image is shown in Figure 1(b). By comparison, it can be clearly seen that the terrain features of the output image are much clearer and more obvious.

#### 3.1.2. Ridge Line Extraction

When extracting ridge lines from a terrain elevation map, we can judge whether a sampling point is a terrain feature point by comprehensively considering multiple profiles after adaptive feature enhancement. The specific process can be divided into five steps, namely, sampling map generation, feature point determination, feature-connected graph construction, ridge line prototype generation, and ridge line smoothing.

**Figure 2:**
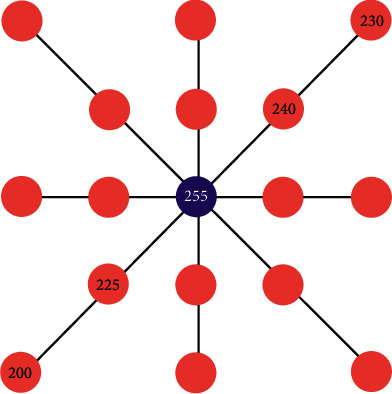
Four profiles with the current sampling point as the center.

**Figure 3:**
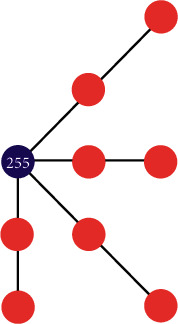
Our method traverses half of the profiles clockwise from the top right direction.

*Step 1*. Sampling map generation: since a terrain elevation map contains many pixels, the input data is sampled with a fixed step size to improve the efficiency of ridge line extraction. Using a method that traverses rows first and then columns, the sampling points are selected in order, and a sampling graph is generated.
*Step 2*. Feature point determination: in the sampling graph, the candidate points are determined by traversing the sampling points from left to right and from top to bottom. Our method traverses four profiles with the sampling point as the center and calculates the height difference between the central sampling point and other sampling points on each profile, as shown in Figure 2. For instance, in the “upper right-lower left” profile, if the elevation value of the central sampling point is higher than other sampling points on this profile, the central sampling point becomes a candidate point for the ridge line.
However, only depending on height differences to determine feature points is problematic since many ridge line feature points in valleys or with a low altitude will also be included. Hence, it is necessary to set a threshold for further screening. The threshold value is calculated based on the global maximum elevation value *I*_max_ and the minimum elevation value *I*_min_ in a terrain elevation map and uses weights to get ridge line feature points. The specific screening process is shown in (3):
  (3) $$\begin{array}{c} P_{\left(x,y\right)}^{\prime }\in \left\{P_{\left(x,y\right)}|H_{\left(x,y\right)}>=w\left(I_{\max}-I_{\min}\right)\right\}, \end{array}$$
where *P*_(*x*, *y*)_ is the previous sampling point before screening; *P*_(*x*, *y*)_′ is the sampled points after screening; *H*_(*x*, *y*)_ is the elevation value of the sampling point; *w* is the weight.
*Step 3*. Feature-connected graph construction: based on the extracted ridge line feature points, a feature-connected graph can be constructed, which can be divided into two steps. Firstly, our method traverses the feature points from left to right and from top to bottom. Afterwards, from the current feature point, our method judges whether there are other feature points on every profile that uses the current feature point as the center. To avoid repeated calculation, only half of the profiles are traversed clockwise, and all feature points on the profile are connected, as shown in Figure 3. After traversing all the feature points, a feature-connected graph can be obtained.
*Step 4*. Ridge line prototype generation: based on the generated feature-connected graph, a ridge line prototype can be generated, which can be divided into three steps. Firstly, a feature edge queue is constructed. In the feature-connected graph, the edge connecting two adjacent feature points is a feature edge, and its weight is the sum of the elevation values of the adjacent feature points. Our method traverses all the feature edges of the feature-connected graph and sorts them based on their weights in descending order to get a feature edge queue. Afterwards, a minimum spanning tree is generated based on the Kruskal algorithm. Finally, our method removes redundant edges by traversing the feature edges in the minimum spanning tree and judging the number of feature edges connected by the two endpoints of the current feature edge. If the number of feature edges connected by an endpoint is less than 2, the current feature edge is redundant and can be removed. After multiple iterative removals, a ridge line prototype is generated.
*Step 5*. Ridge line smoothing: when smoothing ridge lines, our method takes the current feature point as the center and traverses its adjacent feature points. The method accumulates the elevation values and coordinates of all feature points and updates the position of the current feature point according to (4):
  (4) P′x0,y0=∑i=0nHx,iyiPx,iyi∑i=0nHx,iyi,
where *P*′(*x*_0_, *y*_0_) is the updated coordinate of a ridge feature point; *P*(*x*_*i*_, *y*_*i*_) is the coordinate value of all adjacent feature points; *H*(*x*_*i*_, *y*_*i*_) is the elevation value for the feature point. This operation can add some disturbance migrations to the current coordinate point, which can make the extracted ridge lines more realistic.

The process of extracting valley lines is similar to the process of extracting ridge lines. In the stage of the sampling map generation, the sampling map is inversed. The rest of the steps above can be performed to obtain the extraction results of valley lines.

#### 3.1.3. Peak Point Extraction

A peak point is the highest point in a local area of terrain. When dealing with peak points, our method mainly performs the extraction from two perspectives: threshold screening and profile recognition. There are two steps in extracting peak points, namely, threshold calculation and feature point determination.

 
*Step 1*. Threshold calculation: to control the number of candidate peak points, it is necessary to perform a preliminary screening of candidate points by calculating the threshold. Firstly, our method constructs a gray histogram by traversing every gray level on the horizontal axis in reverse order and accumulating the number of pixels under each gray level. When the ratio of the accumulated value to the total number of pixels is *l*, our method stops traversing and takes the grayscale at this moment as the threshold *K*_peak_.
 
*Step 2*. Feature point determination: our method screens the pixels larger than *K*_peak_ in the input elevation map to get candidate points. Since the candidate points only meet the global elevation value, further screening is needed to determine the local optimal feature points, which can be divided into two steps. Firstly, our method sets a profile, which is similar to the process in ridge line extraction. Afterwards, the elevation difference on each profile is calculated clockwise.

After traversing all candidate feature points, the final extraction results of peak feature points can be obtained.

Thus, terrain feature extraction based on adaptive feature enhancement is completed. Based on the steps above, extracted ridge lines, valley lines, and peak points features can be distinguished by different colors and are drawn into a terrain feature map to obtain the final result of terrain feature extraction.

### 3.2. Terrain Elevation Map Generation Based on Self-Attention Mechanism

Sketch-based terrain editing is to generate a terrain elevation map with realistic natural features based on user-edited terrain feature sketches. To generate realistic terrain elevation maps, our method applies a self-attention mechanism to the Conditional Generative Adversarial Network, realizing a terrain elevation map generation algorithm based on self-attention mechanism.

The input of the algorithm is the terrain feature map obtained in Section 3.1 or a terrain feature sketch drawn by a user. Firstly, our method constructs a generator network based on the UNet network, which is used for generating a terrain elevation map. Afterwards, our method constructs a discriminator network based on the PatchGAN network, which is used for distinguishing real terrain samples from generating results. Then, our method adds a self-attention mechanism to the generator and the discriminator, respectively, for capturing global spatial features, which can improve the performance of the generator and the accuracy of the discriminator. Finally, our method trains the discriminator and generator according to the adversarial rules to get a generator model.

#### 3.2.1. Generator Network Construction

In this section, we describe how our method constructs a generator based on a UNet network, whose input and output are both two-dimensional matrices so that our method can generate terrain elevation maps based on terrain feature map inputs from users. In particular, the UNet network we used adopts jumping connections, which can avoid the issue of losing input data feature details due to the increase of network depth.

**Figure 4:**
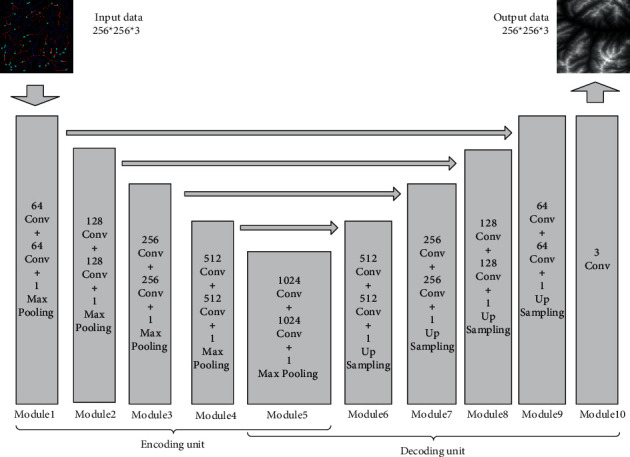
The structure of the generator network.

The input of the generator is a terrain feature map, and the output is a terrain elevation map generated based on prediction. The whole network of the generator consists of 10 modules, which are numbered from 1 to 10, respectively. Specifically, the modules numbered from 1 to 5 consist of two convolutional layers and one pooling layer; the modules numbered from 6 to 9 consist of two convolutional layers and an upsampling layer; the module numbered 10 consists of a convolutional layer. The diagram of the network is shown in Figure 4.

The construction process of the generator network can be divided into three steps, namely, encoding unit construction, decoding unit construction, and jumping connection setup.

*(1) Encoding Unit Construction*. The encoding unit is used for encoding the input data to obtain the color, shape, and local spatial features from the input data. The encoding unit includes module 1 to module 5. Each module consists of two convolutional layers, one pooling layer, and one activation function. Taking module 1 as an example, the construction process includes three steps: convolutional layer construction, pooling layer construction, and activation function setup.

*Step 1*. Convolutional layer construction: the convolutional layer is used to encode the input and obtain the implicit features from the input terrain data. The number of convolutional layers is set to 2; the size of the convolutional kernel is set to 3 × 3; and the number of convolutional kernels is set to 64.
*Step 2*. Pooling layer construction: the pooling layer is used to compress data and reduce the number of parameters so that the overfitting issue can be mitigated. The number of pooling layers is set to 1. The pooling method adopts the maximum pooling, and the pooling layer window size is set to 2 × 2.
*Step 3*. Activation function setup: the activation function is used to activate the input and perform nonlinear output. Leaky ReLU activates the output values that are greater than 0, as shown in equation (5):
  (5) $$\begin{array}{c} \mathrm{LeakyReLU}\left(x\right)=\left\{\begin{array}{cc} x, & x\geq 0\\ ax, & x<0 \end{array}\right.. \end{array}$$

When the input *x* is negative, neurons can still learn, which will not lead to deviations in the final output results. The parameter *a* is generally set to 0.01. Compared with module 1, the number of convolutional kernels of each convolutional layer in modules 2–5 is twice that of the previous module, and other configurations are the same as the previous module.

*(2) Decoding Unit Construction*. The decoding unit is used for decoding the feature information from input and outputting the predicted result. A decoding unit includes five modules, namely, module 6 to module 10. Module 6 to module 9 adopt the combination of two convolutional layers, one upsampling layer, and one activation function. Taking module 6 as an example, the construction process includes three steps: convolutional layer construction, upsampling layer construction, and activation function setup.

 
*Step 1*. Convolutional layer construction: the number of convolutional layers is set to 2; the size of the convolutional kernel is set to 3 × 3; and the number of convolutional kernels is set to 512.
 
*Step 2*. Upsampling layer construction: the upsampling layer and the pooling layer are opposite in terms of functionality; that is, the values of input data are repeatedly filled in the window size area for output. The number of upsampling layers is set to 1, and the window size of the upsampling layer is set to 2 × 2.
 
*Step 3*. Activation function setup: similar to the encoding part, Leaky ReLU is also used for activating output, and the parameter configuration is identical.

Compared with module 6, the number of convolutional kernels of each convolutional layer in module 7 to module 9 is half of that in the previous module, and other configurations are the same as the previous module. Module 10 contains only one convolutional layer; the convolutional kernel size is set to 3 × 3; and the number is set to 1. The module maps the input into the output result of 3 channels and predicts the terrain elevation map.

*(3) Jumping Connection Setup*. Jumping connection can solve the problem that the features of shallow output are gradually lost in subsequent network with the increase of network depth. In Figure 4, the jump connection is indicated by arrows between the corresponding modules in encoding units and decoding units. Taking module 1 and module 9 as an example, the output result of module 1 is directly passed to the third dimension of module 9 input data of the decoding unit so that the shallow output features can be retained in the corresponding deep network structure. This can avoid the loss of feature information and improve the performance of the generator.

#### 3.2.2. Discriminator Network Construction

**Figure 5:**
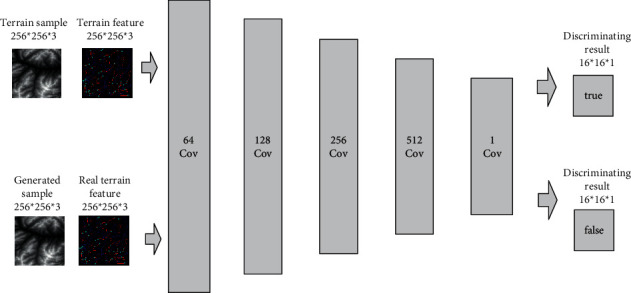
The structure of discriminator network.

Since PatchGAN maps the whole input data as a probability matrix, each value in the matrix represents the discriminant result of the local input. This operation can evaluate the locally generated result and improve the performance of the discriminator. Therefore, this section describes how a discriminator based on PatchGAN is constructed, whose structure is shown in Figure 5.

There are two types of input for the discriminator: (1) real terrain features and terrain samples and (2) real terrain features and generated pseudoterrain samples. Through these two sets of data, the ability to discriminate between real results and generated results can be trained.

The construction of a discriminator network is relatively simple. Firstly, our method sets four convolutional layers, and the convolutional kernel size is set to 4 × 4; the number of convolutional kernels in each convolutional layer is set to 64, 128, 256, and 512, respectively. After each convolutional layer, the output is activated by Leaky ReLU. Then, our method sets a convolutional layer, where the convolutional kernel size is set to 1 × 1, and the number is set to 1, which is used for combining the number of input channels into 1. Afterwards, the output is activated by a Sigmoid function to obtain a binary prediction result of the local area, where the size of the output probability matrix is set to 16 × 16, and the number of channels is set to 1. Finally, our method accumulates all the values in the probability matrix and calculates the average value to obtain the final discrimination result.

#### 3.2.3. Self-Attention Mechanism Construction

In the process of terrain generation, the input terrain features include not only color, shape, and local space but also global spatial features. In real terrain elevation map samples, ridge line and valley line feature areas are often continuous areas, which are all global spatial features. This section introduces a self-attention mechanism to capture the global spatial features of the input data.

**Figure 6:**
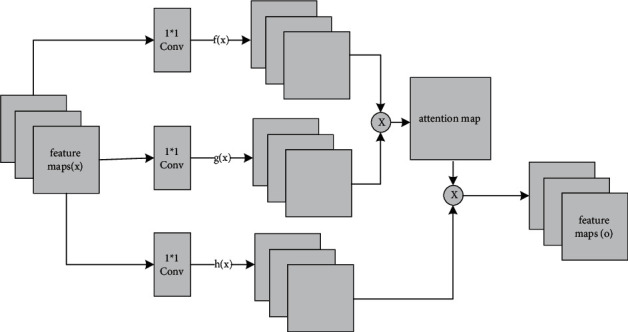
The flow of self-attention calculation.

The process of building a self-attention module is shown in Figure 6. The input data is the feature map output from the previous layer, and the shape of the input data is [*h*, *w*, *c*], where the length of the input feature map is *h*, the width is *w*, and the number of channels is *c*. The output is the fusion result of the self-attention feature and the original feature map, whose shape is consistent with the input data. The construction process includes three steps: input data initialization, local self-attention feature map calculation, and global self-attention feature map calculation.

*(1) Input Data Initialization*. The input data is initialized by convolution. As shown in equation (6), our method uses convolutional kernels *W*_*f*_ and *W*_*g*_ with a number of *c*/*k* and a size of 1 × 1 to perform convolution operations on the input data to obtain feature maps *f*(*x*) and *g*(*x*). In addition, our method uses a convolutional kernel *W*_*h*_ with a number of *c* and a size of 1 × 1 to perform convolution operation on the input data to obtain feature maps *h*(*x*).

(6) $$\begin{array}{c} f\left(x\right)=W_{f}x,\\ g\left(x\right)=W_{g}x,\\ h\left(x\right)=W_{h}x, \end{array}$$

where the shape of *f*(*x*) and *g*(*x*) is [*h*, *w*, *c*/*k*] and the shape of *h*(*x*) is [*h*, *w*, *c*].

*(2) Local Self-Attention Feature Map Calculation*. Our method first readjusts the shapes of feature maps *f*(*x*), *g*(*x*), and *h*(*x*) to [*h∗w*, *c*/*k*], [*h∗w*, *c*/*k*], and [*h∗w*, *c*], respectively. Afterwards, our method calculates *f*(*x*)*g*(*x*)^*T*^ according to equation (7):

(7) $$\begin{array}{c} a_{j,i}=\frac{\exp\left(s_{i,j}\right)}{\sum_{i=1}^{N}\exp\left(s_{i,j}\right)},s_{i,j}=f\left(x_{i}\right)g{\left(x_{j}\right)}^{T}. \end{array}$$

Then, the softmax function is used to activate the output of the calculation result and obtain a local self-attention feature map *a*_*j*,*i*_ with a shape of [*h∗w*, *h∗w*], which represents the local attention to the *i*-th pixel when the *j*-th pixel is generated.

*(3) Global Self-Attention Feature Map Calculation*. The global self-attention feature map is obtained by fusing the global spatial information and the local self-attention feature map, as shown in equation (8):

(8) $$\begin{array}{c} o_{j}=\sum_{i=1}^{h*w}a_{j,i}h\left(x_{i}\right)\;,h\left(x_{i}\right)=W_{h}x_{i}, \end{array}$$

where *a*_*j*,*i*_ represents the local self-attention feature map; *h*(*x*_*i*_) represents the global spatial information; and *o*_*j*_ represents the global self-attention feature map when the *j*-th region is generated, and the shape is [*h∗w*, *c*].

In order to control the influence of the self-attention feature map, it is necessary to set parameters for the self-attention feature map and then fuse it with the initial input as the output. In this case, the network structure can first rely on local spatial features and then rely on remote spatial features during the training process, as shown in equation (9):

(9) $$\begin{array}{c} y_{j}=\gamma o_{j}+x_{j}, \end{array}$$

where *γ* is the weight of self-attention with an initial value of 0. *γ* can be gradually assigned a larger value during the training process. The self-attention module described in this section is added after module 9 of the generator and after the fourth convolutional layer of the discriminator, and the construction of the entire network is completed.

#### 3.2.4. Network Training

We train the network after the network is built. The training of the network mainly includes three steps, namely, optimization target determination, training process setup, and loss function construction.

*(1) Optimization Target Determination*. The goal of this step is to generate a real terrain elevation map, and the evaluation standard is to judge whether it is a terrain elevation map generated by the generator or a real terrain elevation map sample. Since this is a binary classification problem, we adopt a binary classification cross-entropy loss function to optimize the target. The calculation of the binary classification cross-entropy is shown in equation (10):

(10) $$\begin{array}{c} L_{n}=-\frac{1}{n}\sum_{i=1}^{n}\left[y_{i}\log\left({\hat{y}}_{i}\right)+\left(1-y_{i}\right)\left(1-\log\left(1-\hat{y}\right)\right)\right], \end{array}$$

where *n* is the number of samples; *i* is the current *i*-th sample; *y*_*i*_ is the label of *i*-th sample; ${\hat{y}}_{i}$ is the probabilistic predicted value of *i*-th sample.

*(2) Training Process Setup*. The training process of a Generative Adversarial Network is a confrontation between generator and discriminator. The process of confrontation training is to fix the generator first, where the weights of the discriminator are trained and updated so that the ability of the discriminator to distinguish between true and false images can be maximized. Then the discriminator is fixed, where the network weights of the generator are updated so that the ability of the discriminator to distinguish between true and false images is minimized. As the generator cannot distinguish the true graph from the false graph, the performance of the generator can be improved. These processes are executed alternately until the loss function converges; the performance of the generator will be optimal at this moment, which can be used in practical applications.

*(3) Loss Function Construction*. According to the optimization goal and training process, the confrontation loss can be obtained, as shown in equation (11):

(11) $$\begin{array}{c} \underset{G}{\min}\underset{D}{\max}L_{\mathrm{G,D}}=E_{\left(x,y\right)}\left[\mathrm{logD}\left(G\left(x|y\right)\right)\right]+E_{\left(x,z\right)|y}\left[\log\left(\mathrm{1-}D\left(G\left(z|y\right)\right)\right)\right], \end{array}$$

where *G* is the generator; *D* is the discriminator; *x* is the real terrain data; and *z* is the noise data randomly generated in the generator. In addition, in order to measure the difference between the generated results and the official data at the pixel level and further improve the performance of the generator, this paper also introduces L_1_ loss, as shown in equation (12):

(12) $$\begin{array}{c} L_{1}=E_{\left(x,y\right)}\left[{\left\|G\left(z|y\right)-G\left(x|y\right)\right\|}_{1}\right]. \end{array}$$

Finally, the total loss function is obtained by synthesizing the above-mentioned confrontation loss and L_1_ loss, as shown in equation (13):

(13) $$\begin{array}{c} L=L_{G,D}+L_{1}. \end{array}$$

In this way, the construction of the whole network is completed. According to the training rule, we train the network weights and optimize the performance of the generator so that the trained generator can be used in the task of generating a terrain elevation map.

### 3.3. 3D Terrain Visualization Based on Levels of Detail

In order to build terrain mesh efficiently and ensure the smoothness of terrain visualization, this section studies the 3D terrain visualization algorithm based on Levels of Detail. Firstly, the nodes are defined by a quadtree structure, and then the terrain mesh is constructed according to the node information. Finally, multiple textures are added to the mesh.

#### 3.3.1. Quadtree Structure Construction

Quadtree structure is a tree structure where each node includes 0 or 4 subnodes. When each node is split, the details can be improved. For a terrain mesh, when the subdivision degree of the mesh is increased, the details of the mesh will be richer. The construction of a quadtree structure can be divided into the following steps: defining nodes, calculating node coordinates, and judging whether a node needs to be divided and stored, and a complete quadtree structure can be obtained finally.

#### 3.3.2. Terrain Mesh Construction

Based on the quadtree structure, a terrain mesh with multiple Levels of Detail can be created. Since a terrain mesh is composed of triangular faces that are surrounded by vertices, there are two steps to construct a terrain mesh, namely, creating vertices and drawing triangular faces. The drawing of triangle faces is based on the quadtree structure, where the nodes are recursively operated from the root node until all elements in the quadtree node array are traversed. Thus, a terrain mesh with Levels of Detail is constructed.

#### 3.3.3. Multiple Texture Addition

Since 3D terrain visualization needs both terrain mesh and texture, it is necessary to add multiple textures to improve the realism of visualization results. It can be divided into three steps, namely, selecting terrain textures, creating mask textures, and fusing output. As shown in Table 1, there are four kinds of terrain textures used for visualization, namely, land, grassland 1, grassland 2, and moss, which correspond to different elevation value intervals in terrain, respectively. When performing texture mapping, the texture pixel value of a mesh vertex is calculated based on the value of mask texture. The four channel values in the mask texture and the corresponding pixel values in the four textures are multiplied and then summed, and the fused texture is the final output.

In this way, the 3D terrain visualization based on Levels of Detail is completed, which can realize the efficient construction of terrain mesh and realistic texture mapping, making interactive terrain editing more interesting.

## 4. Experimental Analysis

The experiment was designed to verify the feasibility and effectiveness of the multifeature sketch terrain elevation map generation method based on self-attention mechanism. The hardware environment of the verification system includes AMD Ryzen 7 4800H CPU, 16 GB RAM, and NVIDIA GeForce RTX2060 GPU. The software environment includes Windows 10 operating system, PyCharm 2019, Visual Studio 2019, and Unity3D 2019. *Python* and C# are used as the main development languages.

In this study, six experiments are designed, namely, terrain feature extraction experiment, terrain generation comparative experiment, self-attention mechanism comparative experiment, terrain mesh construction comparative experiment, multiple textures addition experiment, and interactive terrain editing software experiment.

### 4.1. Experiment for Terrain Feature Extraction

To verify the feasibility of terrain feature extraction based on adaptive feature enhancement, a terrain feature extraction experiment was designed.

**Figure 7:**
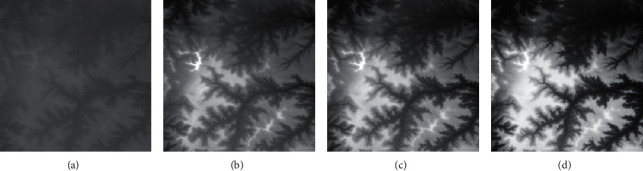
Comparison of original input data and enhanced data. (a) Input of a terrain elevation map. (b) Result when *K*_gray_ is set to 0.05%. (c) Result when *K*_gray_ is set to 1%. (d) Result when *K*_gray_ is set to 1.5%.

Firstly, adaptive feature enhancement was performed. Taking the terrain elevation map input in Figure 7(a) as an example, data preprocessing and grayscale expansion were performed. When expanding grayscale, *K*_gray_ is set to different values in Figures 7(b)–7(d). It can be seen when *K*_gray_ is set to 1%, the details of terrain features can be well retained while the features can be enhanced. According to the result in Figure 7(c), terrain feature extraction was performed.

The specific steps of ridge line extraction are as follows.

**Figure 8:**
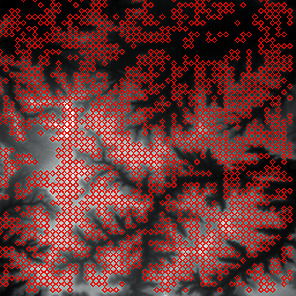
The candidate feature points for ridge lines obtained by profile recognition and screening.

**Figure 9:**
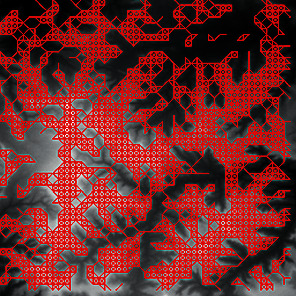
Feature-connected graph build based on feature points.

**Figure 10:**
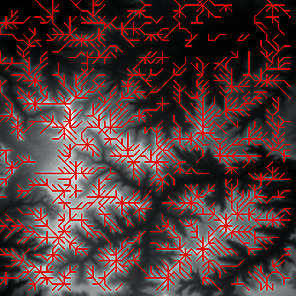
The minimum spanning tree extracted from feature-connected graph.

**Figure 11:**
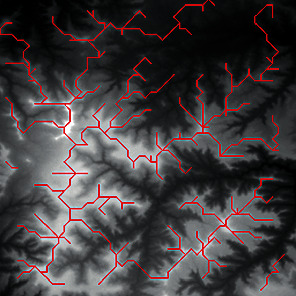
Feature line prototype after redundant edge removal.

**Figure 12:**
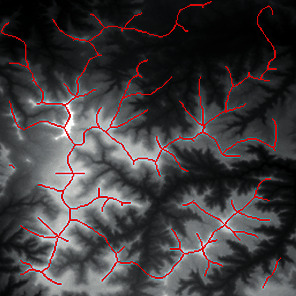
The final result of ridge line feature extraction.

First, we set the sampling distance to 5 pixels to generate a sampling map. We also set the profile for the sampling points, where the length of the profile was set to 7, making preliminary screening based on the profile. Then, according to equation (3), the threshold was set for further screening. Based on our pilot study, we found that 0.03 was an ideal value for the weight *w*. The final result of feature point extraction is shown in Figure 8. Afterwards, the feature-connected graph was constructed by traversing all feature points, starting from the current feature point and connecting the feature points on the profile. The final construction result is shown in Figure 9. Then, we sorted the feature edges and stored them in the feature edge queue to create a minimum spanning tree according to the feature edge queue, as shown in Figure 10. The number of iterative deletions was set to 3, so the redundant edges in the minimum spanning tree can be removed to obtain a prototype of the ridge feature line, as shown in Figure 11. Then, smoothing was performed on the feature-connected graph to make the feature lines more similar to real terrain features. The smoothing result is shown in Figure 12.

**Figure 13:**
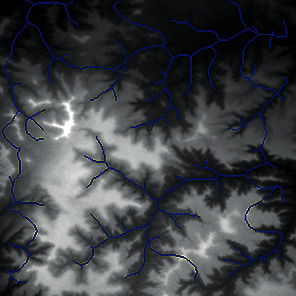
The result of valley line feature extraction.

The valley line extraction process is similar to the ridge line extraction process. When generating a valley line sampling map, the sampling map is inversed, and then the steps above are performed to obtain the valley line extraction result, as shown in Figure 13.

**Figure 14:**
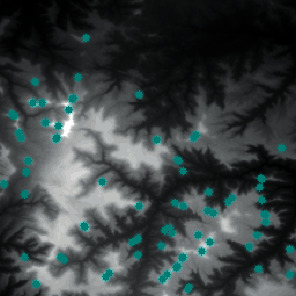
The result of peak point feature extraction.

Finally, we extracted the peak points. First, we calculated the threshold value. Our pilot study showed that when the accumulated number of pixels accounted for 5% of the total number of pixels, the number of selected peak points was more suitable. Then, we determined the feature points and the peak points through the profile recognition and elevation difference. The extraction result of the peak point is shown in Figure 14.

### 4.2. Comparative Experiment for Terrain Generation

To verify the feasibility of terrain elevation map generation based on a self-attention mechanism, this experiment was performed from two aspects: (1) using real terrain features to demonstrate generated terrain elevation maps and compare the differences between the generated results and real terrain samples; (2) using hand-painted terrain features to demonstrate generated terrain elevation maps and 3D visualization effects.

*(1) Using Real Terrain Features*. We input real terrain features in the trained generator model for prediction and compared the differences between the generated terrain elevation maps and real terrain samples.

The experimental results are shown in Table 2, where the features of ridge lines, valley lines, and peak points in the generated terrain elevation maps were consistent with the features in real terrain feature maps. To evaluate the similarity between the generated results and the real terrain results, the SSIM image similarity evaluation algorithm was used. The calculation result of SSIM is between 0 and 1; the closer the result is towards 1, the more similar the two images will be. It can be seen that the SSIM results obtained by our method were all greater than 0.5, which suggested that the similarity with the original image was high. This demonstrated the feasibility of our proposed method, which can meet the needs of interactive terrain editing.

*(2) Using Hand-Painted Terrain Features*. To further verify the feasibility of our proposed method, this experiment input terrain feature sketches drawn by users into the trained generator model for prediction and demonstrated the generated terrain elevation maps and 3D visualization results.

The experimental results are shown in Table 3. It can be seen that terrain feature sketches can be very simple, which only have a few simple strokes. We input these sketches into the trained generator model, which can generate highly realistic terrain elevation maps based on these sketches.

We also used World Machine software to render the terrain elevation maps based on user sketches to further verify the realism of editing results. It can be seen that the rendered terrain results have realistic ridge lines, valley lines, and peak points, which can meet the demands of interactive editing.

### 4.3. Comparative Experiment for Self-Attention Mechanism

To verify the effectiveness of the self-attention mechanism in terrain elevation map generation, this experiment compared the results with and without the self-attention mechanism.

The input was the terrain features of real samples, as shown in Table 4. It can be seen that some ridge line and valley line structures in the generated results are incomplete without a self-attention mechanism, as shown in the places marked by red circles. In contrast, the results with the self-attention mechanism are much better in terms of completeness and detail, and the generator model also has a higher performance.

### 4.4. Comparative Experiment for Terrain Mesh Construction

To verify the feasibility and effectiveness of the terrain mesh construction in our proposed method, a terrain mesh construction experiment based on Levels of Detail was designed.

**Figure 15:**
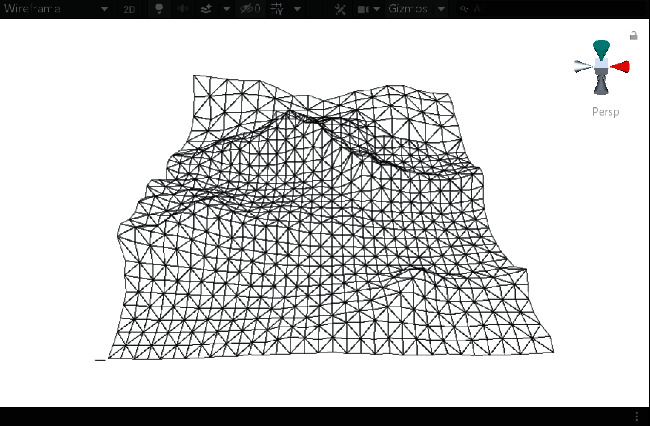
The result of terrain mesh constructed based on Levels of Detail.

First, we established a quadtree structure. Afterwards, we constructed a terrain mesh and a vertex array. According to whether each node can be divided, a terrain mesh was constructed by drawing triangle faces. In the scene view in Unity3D editor, WireFrame mode was used to display the terrain mesh. The result is shown in Figure 15.

It can be seen that the terrain mesh constructed based on Levels of Detail has lower mesh complexity. While the areas that are far from the viewpoint have lower details, the areas that are close to the viewpoint have greater details. By moving the viewpoint, the changes of details can be clearly seen in local terrain areas. As shown in Table 5, when the camera moved towards the terrain mesh, the terrain details became gradually enriched. Thus, our method realized dynamic control of mesh details, and rendering efficiency was high.

### 4.5. Experiment for Multiple Texture Mapping

To verify the effectiveness of using multiple textures to enhance the realism of 3D terrain, an experiment of multiple texture mapping was designed.

First, a mask texture was created based on a terrain elevation map drawn by a user. Then, the sample texture and mask texture were fused for outputting multiple textures, which adopted different elevation value intervals for different texture mapping. Finally, random translations and mirror operations were performed on the sample texture to reduce texture repetitions during the tiling process.

**Figure 16:**
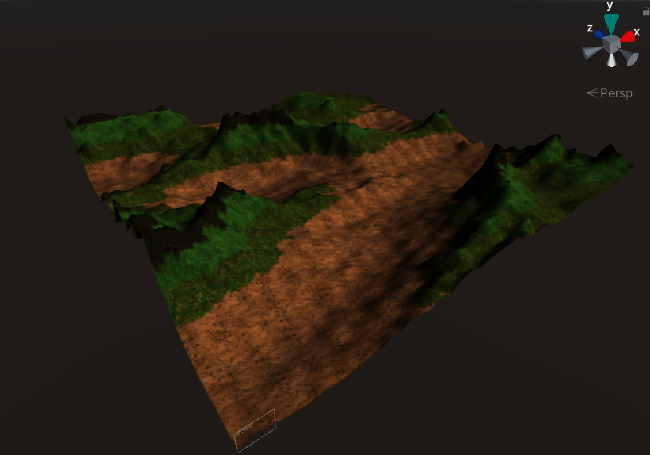
The result of adaptive texture mapping.

The experimental results are shown in Figure 16. It can be seen that multiple texture mapping can assign different textures based on the elevation value intervals of the terrain elevation map. From 3D visualizations of terrains, users can have an intuitive and fun experience in interactive terrain editing and terrain elevation map creation.

### 4.6. Experiment for Interactive Terrain Editing

To provide a good user experience for interactive terrain editing, we designed an interactive terrain editing tool based on the methods for terrain feature extraction, terrain elevation map generation, and 3D terrain visualization that are described earlier in this paper. With the help of this tool, users can freely draw ridge line, valley line, and peak point features on a sketch. The tool can generate terrain elevation maps with realistic features in real time based on the sketch and provide 3D visualization for the terrain that users can interact with.

The interactive terrain editing tool based on a multifeature sketch can be divided into four modules: terrain feature extraction module, terrain elevation map generation module, terrain visualization module, and human-computer interaction module. Specifically, terrain feature extraction is used for extracting terrain features, creating data sets, and training networks to get the generator model with the best performance. With this generator model, the terrain elevation map generation module can generate a terrain elevation map based on a terrain feature sketch drawn by a user. The terrain visualization module adopts LOD mode to build a terrain mesh and adopts multiple texture mapping to improve the realism of 3D terrain. Finally, the human-computer interaction module provides a user interface for the functionalities above, creating a good user experience for the interactive terrain editing tool.

*(1) Terrain Feature Extraction Module*. Terrain feature extraction module is the core module for interactive terrain editing. In the terrain editing tool, terrain features are distinguished by using different colors and drawing methods. Specifically, red lines are used for ridge lines; blue lines are used for valley lines; cyan dots are used for peak points.

*(2) Terrain Elevation Map Generation Module*. Terrain elevation map generation module is the basis for real-time interactive terrain editing. The trained generator model can be loaded by the LoadModel() method in the Keras library. A series of format conversions are performed on the user-created feature sketch to obtain a suitable data format for the model input, and the Predict() method is called to predict and generate a terrain elevation map. Finally, the generated result is converted to PNG format, where the user can choose different resolutions to save the file.

*(3) Terrain Visualization Module*. Terrain visualization module allows users to observe the generated results intuitively. The module includes two submodules: mesh construction and texture mapping. Due to the LOD-based mesh construction, it is possible to ensure the user experience on low-performance devices.

*(4) Human-Computer Interaction Module*. Human-computer interaction module can have a great impact on user experience for the terrain editing tool. The module can be divided into two submodules: interactive user interface module and interactive function module. Specifically, the interactive user interface module includes a main user interface, a feature extraction user interface, and a roaming user interface; interactive function module includes terrain feature extraction function, editing and saving function, and roaming display function.

**Figure 17:**
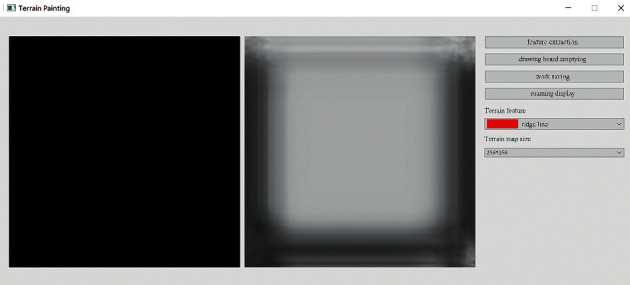
The initial user interface of our interactive terrain editing tool.

The initial user interface of our interactive terrain editing tool is shown in Figure 17. The black area on the left side is the user sketch drawing area, and the white area on the right side is the terrain generation area. The rightmost sidebar has six functions, namely, feature extraction, drawing board emptying, work saving, roaming display, terrain feature selection, and output resolution selection.

**Figure 18:**
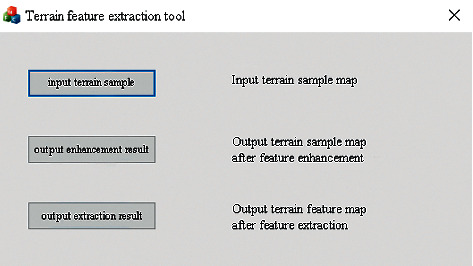
The interface of terrain feature extraction.

**Figure 19:**
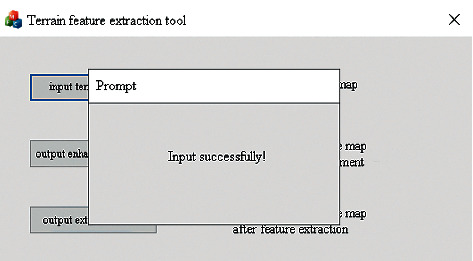
The interface for successful input.

**Figure 20:**
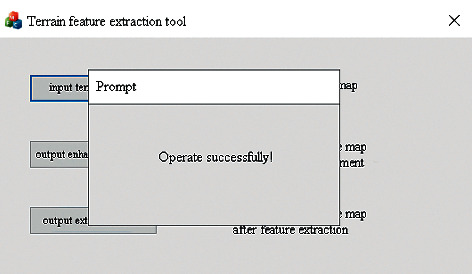
The interface for successful feature enhancement and feature extraction.

Users can click the feature extraction button to start terrain feature extraction, which can perform feature enhancement and feature extraction operations on the local terrain elevation map data sets. The user interface for terrain feature extraction is shown in Figure 18. After successful input, feature enhancement, and extraction, a popup will be displayed to indicate the operation is successful, as shown in Figure 19 and Figure 20.

**Figure 21:**
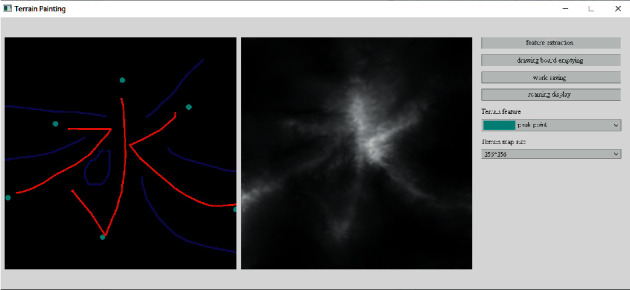
The generated terrain elevation map based on a user sketch.

Users can select different brushes to draw terrain features on a sketch, and the generated results will be displayed in the terrain generation display area. As shown in Figure 21, three types of features, including ridge lines, valley lines, and peak points, are drawn on the sketch. Since the low-level GPU-driven generator is used, the calculation can be done in real time. When a user is drawing terrain features on the sketch, the prediction of terrain elevation map can be completed in real time.

**Figure 22:**
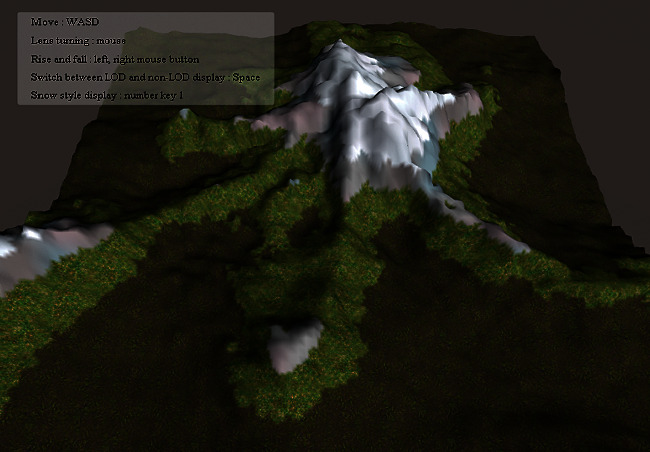
The result of snow-style terrain visualization.

After drawing, the user can click the roaming display button to start interactive roaming to observe the visualization effect of 3D terrain. To increase the fun for user interaction, users can click the number key 1 to display a snow scenery, which can display a terrain under different weather conditions, as shown in Figure 22.

## 5. Conclusions and Future Work

To address the issues of low efficiency in obtaining terrain feature map by manual annotation and poor realism in generated terrain elevation map, this paper proposes a method of generating terrain elevation map based on self-attention mechanism and multifeature sketch. First, a terrain feature extraction method based on adaptive feature enhancement and profile recognition is proposed to realize the rapid and automatic terrain feature extraction of ridge lines, valley lines, and peak points, which can perform well in large-scale terrain feature map generations. Afterwards, our method adopts terrain elevation map generation based on the self-attention mechanism, where a generator network based on UNet network and a discriminator network based on PatchGAN network are built, and then self-attention mechanism are added to the generator and the discriminator, respectively, to capture the global spatial features for generating a realistic terrain elevation map. Finally, by constructing a terrain mesh and adding multiple textures, a 3D terrain visualization method based on Levels of Detail is proposed, which can provide high rendering efficiency and realistic visualization results. In this paper, an interactive terrain editing tool based on a multifeature sketch is implemented, which allows users to interact with the generated terrain. Experimental data show that the proposed method is effective in terrain feature extraction, terrain elevation map generation, and 3D visualization. In addition, the editing tool can run smoothly, providing intuitive user interactions and a good user experience.

Since the research on interactive terrain editing techniques is conducted under specific conditions, there are certain limitations and spaces for optimization. Here are a few points that can be further studied in theory and practice:

1. *Network Structure Optimization*. The network adopted in this paper is based on the Conditional Generative Adversarial Network structure. In the future, we can explore the influence of different network structures on the generation results and the efficiency of different training models and further optimize the network structure.
2. *Texture Mapping Optimization*. Despite the fact that multiple texture mapping is used in this paper, there is a limit on the number of textures. In the future, we can explore new ways to create mask textures, eliminating the limitation on the number of sample textures to achieve more realistic texture mapping.

## Figures and Tables

**Table 1:** Textures used in the experiment.

Land	Grassland 1	Grassland 2	Moss
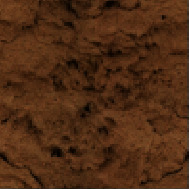	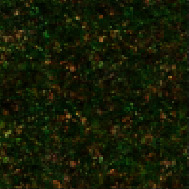	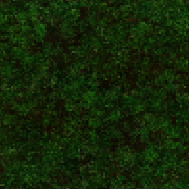	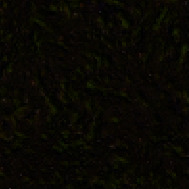

**Table 2:** Generated terrain results based on real terrain features.

Terrain features	Real terrain samples	Generated results	SSIM
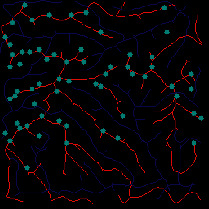	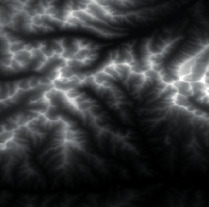	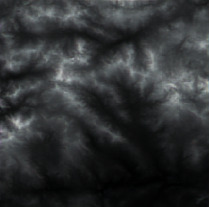	0.60
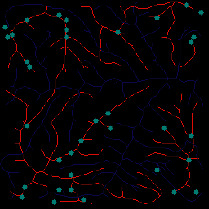	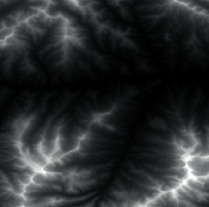	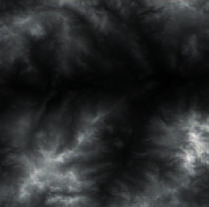	0.65
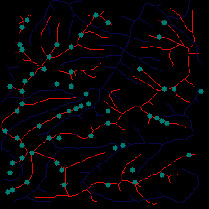	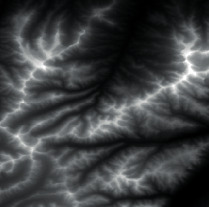	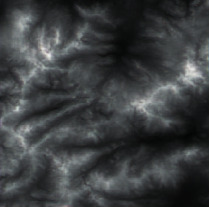	0.63

**Table 3:** Generated terrain results based on sketch input.

Terrain feature sketches	Generated results	Rendered results
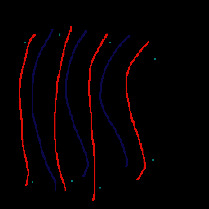	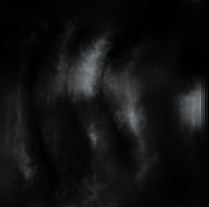	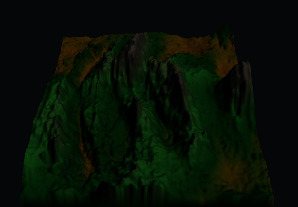
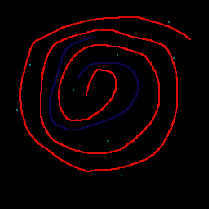	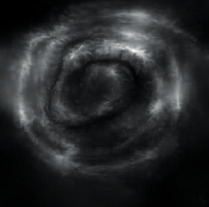	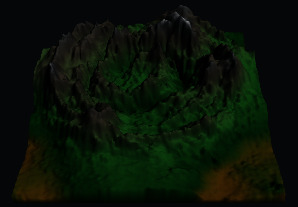
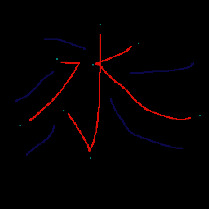	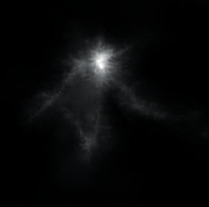	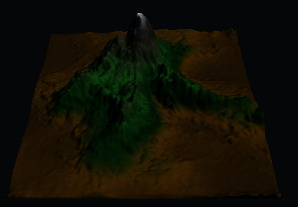

**Table 4:** Comparison of generated terrain elevation maps with and without self-attention mechanism.

Terrain features	Real terrains	Without self-attention mechanism	With self-attention mechanism
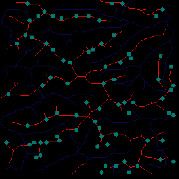	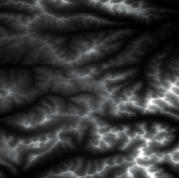	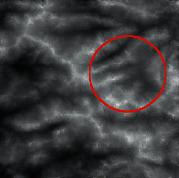	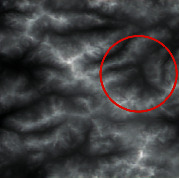
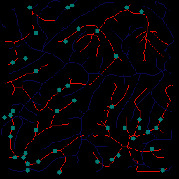	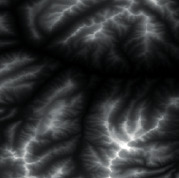	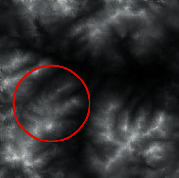	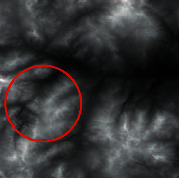
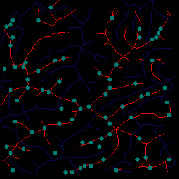	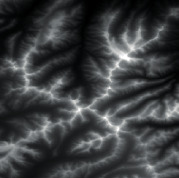	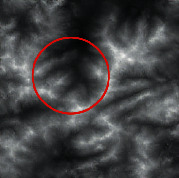	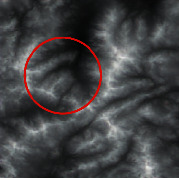

**Table 5:** Generated terrain meshes based on different distances.

Distance = 9000	Distance = 6000
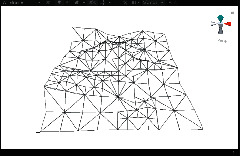	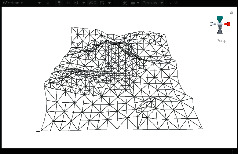
Distance = 3000	Distance = 1000
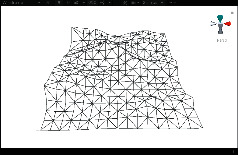	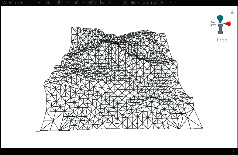

## Data Availability

The data used to support the findings of this study are available from the corresponding author upon request.

## References

[1] Song H. C., Gao Y., Wei Y. M. (2004). Interactive generation and representation of three-dimensional terrain. *Computer Engineering and Applications*.

[2] Yin H. F., Zheng C. W., Hu X. H. (2012). Interactive digital terrain synthesis algorithm. *Journal of Computer-Aided Design and Graphics*.

[3] Zeng Y. Y., Kang F. J., Yang H. Z. (2013). Adaptive multi-feature fusion for fast realistic terrain mapping. *” Journal of Image and Graphics*.

[4] Liu Y., Tou X. G., Li H. L. (2016). An efficient parallel method for topography generation by Perlin noise. *Bulletin of Science and Technology*.

[5] Guérin É., Digne J., Galin E. (2017). Interactive example-based terrain authoring with conditional generative adversarial networks. *ACM Transactions on Graphics*.

[6] Cordonnier G., Cani M.-P., Benes B., Braun J., Galin E. (2018). Sculpting mountains: interactive terrain modeling based on subsurface geology. *IEEE Transactions on Visualization and Computer Graphics*.

[7] Jensen A. W., Rant N. N., Møller T. N., Billeskov J. A. Deep convolutional generative adversarial network for procedural 3D landscape generation based on DEM.

[8] Paris A., Galin E., Peytavie A., Guérin E., Gain J. (2019). Terrain amplification with implicit 3D features. *ACM Transactions on Graphics*.

[9] Fischer R., Dittmann P., Weller R., Zachmann G. (2020). AutoBiomes: procedural generation of multi-biome landscapes. *The Visual Computer*.

[10] Chang Y.-C., Song G.-S., Hsu S.-K. (1998). Automatic extraction of ridge and valley axes using the profile recognition and polygon-breaking algorithm. *Computers & Geosciences*.

[11] Huang P., Liu Z. (2005). Extraction of ridge and valley lines based on terrain gradient direction. *Journal of Wuhan University (Natural Science Edition)*.

[12] Zhang H., Liu Y., Ma Z. (2015). A terrain skeleton feature extraction method based on morphological coding. *Computer Research and Development*.

[13] Zou K., Wong H., Li W. (2017). DEM terrain feature line extraction with controllable saliency. *Journal of Image and Graphics*.

[14] Goodfellow I. J., Pouget-Abadie J., Mirza M. Generative adversarial networks.

[15] Mirza M., Osindero S. (2014). Conditional generative adversarial nets. *Computer Science*.

[16] Radford A., Metz L., Chintala S. Unsupervised representation learning with deep convolutional generative adversarial networks.

[17] Isola P., Zhu J. Y., Zhou T., Efros A. A. Image-to-Image translation with conditional adversarial networks.

[18] Zhang H., Goodfellow I., Metaxas D., Odena A. Self-attention generative adversarial networks.

[19] Clark J. H., James H. (1976). Hierarchical geometric models for visible surface algorithms. *Communications of the ACM*.

[20] Wang Z., Lü X. (2018). Terrain rendering LOD algorithm based on improved restrictive quadtree segmentation and variation coefficient of elevation. *Journal of Beijing Institute of Technology (Social Sciences Edition)*.

[21] Du D., Gao B. L., Tian L. (2020). Combining fast layer-based DCT and dynamic LOD for terrain compression mapping techniques. *Computer Engineering and Applications*.

[22] Zhao R., Zhang Y., Wang J. (2012). Multi-map terrain texture synthesis algorithm under feature control. *Computer Engineering*.

[23] Gao B., Zhang B., Dou M. (2018). Multi-resolution texture seamless mapping based on error control. *Computer Engineering and Design*.

[24] Zhou Y., Li J., Xu W. (2018). A real-time beach scene simulation based on Poisson fusion. *Journal of Wuhan University (Natural Science Edition)*.

